# Heavy metal pollution and ischemic stroke: multimechanistic pathogenesis and countermeasures

**DOI:** 10.3389/fpubh.2025.1650999

**Published:** 2025-08-29

**Authors:** Junge Liu, Lin Wu

**Affiliations:** Department of Cardiology, Shengjing Hospital, China Medical University, Shenyang, China

**Keywords:** heavy metal pollution, ischemic stroke, oxidative stress, environmental exposure, public health intervention

## Abstract

Heavy metal pollution is a significant environmental risk factor that profoundly impacts cerebrovascular health, particularly in the pathophysiology of ischemic stroke. This article outlines the relationship between metal exposure and stroke risk, highlighting regional differences potentially caused by contaminated food chains and industrial processes. We provide an in-depth discussion on the complex roles of lead (Pb), cadmium (Cd), arsenic (As), mercury (Hg), copper (Cu), and zinc (Zn) in the pathophysiology of stroke, with a particular focus on five key mechanisms: redox imbalance, neurotransmitter dysregulation, neuroinflammation, endothelial dysfunction, and coagulation disorders. Additionally, the review summarizes recent targeted therapeutic strategies for heavy metals, including antioxidants, metal chelators, inflammasome inhibitors, and epigenetic modifications, which show promise in neuroprotection. Research indicates that these strategies offer new perspectives for precision medicine in stroke treatment. We emphasize the importance of considering environmental factors in stroke prevention and advocate for pollution reduction as a means to improve public health. This review integrates molecular neuroscience and environmental toxicology, providing new insights and potential solutions to address the cerebrovascular diseases associated with heavy metals. These findings not only enhance our understanding of stroke’s pathophysiological mechanisms but also lay the foundation for future clinical treatment and prevention strategies.

## 1 Introduction

Heavy metal pollution has emerged as a global environmental and public health crisis, driven by anthropogenic activities such as industrial emissions, agricultural practices, and improper waste disposal. These activities contribute to widespread contamination of soil, water, and air (1), with disparate regional exposure patterns directly shaping population-level cerebrovascular health disparities. Recent studies have shown that exposure to heavy metals can adversely affect human health by inducing oxidative stress, inflammation, and coagulation dysfunction (2–4). Long-lasting contaminants in food pose a subtle but significant threat to human health, particularly through their association with cardiovascular diseases (CVDs), such as ischemic stroke, which remains the leading cause of death and disability worldwide (5, 6).

Ischemic stroke, recognized as a critical component of the CVD continuum, arises from thromboembolic cerebrovascular events and shares pathomechanistic foundations with hypertension, atherosclerosis, and systemic inflammation through common pathways (7). Emerging epidemiological evidence establishes dose-dependent associations between chronic heavy metal exposure (particularly blood lead, cadmium, arsenic, and urinary cadmium) and stroke incidence, with distinct regional exposure paradigms—including southern Taiwan (China) and southern China (8, 9), where industrial and occupational pollution as well as dietary exposure contribute to increased stroke risk. Notably, in 2021, global stroke deaths attributable to Pb exposure reached approximately 556,600, with ischemic stroke accounting for the highest age-standardized mortality rate (3.21 per 100,000 population) among all stroke subtypes (8). Regionally, cadmium (Cd) contamination in rice cultivation in southern China has exacerbated the burden of stroke, while in South Asia, As contamination in groundwater irrigation and the use of Cd-rich phosphate fertilizers have led to sustained exposure of major crops to these harmful substances (9, 10). These cases show pollution-driven food chain amplification turns heavy metals into insidious, population-wide threats to cerebrovascular health, highlighting the need to reassess environmental-cerebrovascular interplay amid industrialized agricultural pollution’s growing global impact.

Although existing studies have established a significant association between ischemic stroke and heavy metal pollution, the mechanisms underlying this relationship remain inadequately elucidated. Current research primarily focuses on oxidative stress and endothelial dysfunction induced by heavy metals. However, more research is needed to determine the precise roles that these factors play in the pathophysiology of ischemic stroke, particularly with regard to neurovascular unit responses and post-ischemic molecular mechanisms (11). Five major pathogenic mechanisms have been identified based on current scientific evidence: (1) redox imbalance, (2) inflammatory immune activation, (3) endothelial injury, (4) neurotransmitter dysregulation, and (5) abnormalities in platelet hyperactivation and coagulation.

To effectively address the pathogenic impact of heavy metals, novel therapeutic strategies have been proposed, including multi-target interventions such as antioxidants (12), metal chelators (13), inflammasome inhibitors (14), and epigenetic modifications (15). With the aid of modern nanoparticle delivery systems, these strategies significantly enhance the efficiency of drugs crossing the blood–brain barrier (BBB). Furthermore, the multi-mechanism synergistic approach not only improves therapeutic efficacy but also increases the flexibility and adaptability of treatment plans.

This review, from the perspectives of molecular neurobiology and environmental toxicology, suggests innovative strategies for combating pollution-induced cerebrovascular diseases. It advocates for integrating the concept of planetary health into stroke prevention, offering theoretical support for the management of heavy metal-related strokes and advancing the field of cerebrovascular disease prevention.

## 2 The association between heavy metal exposure and stroke

**Figure 1:**
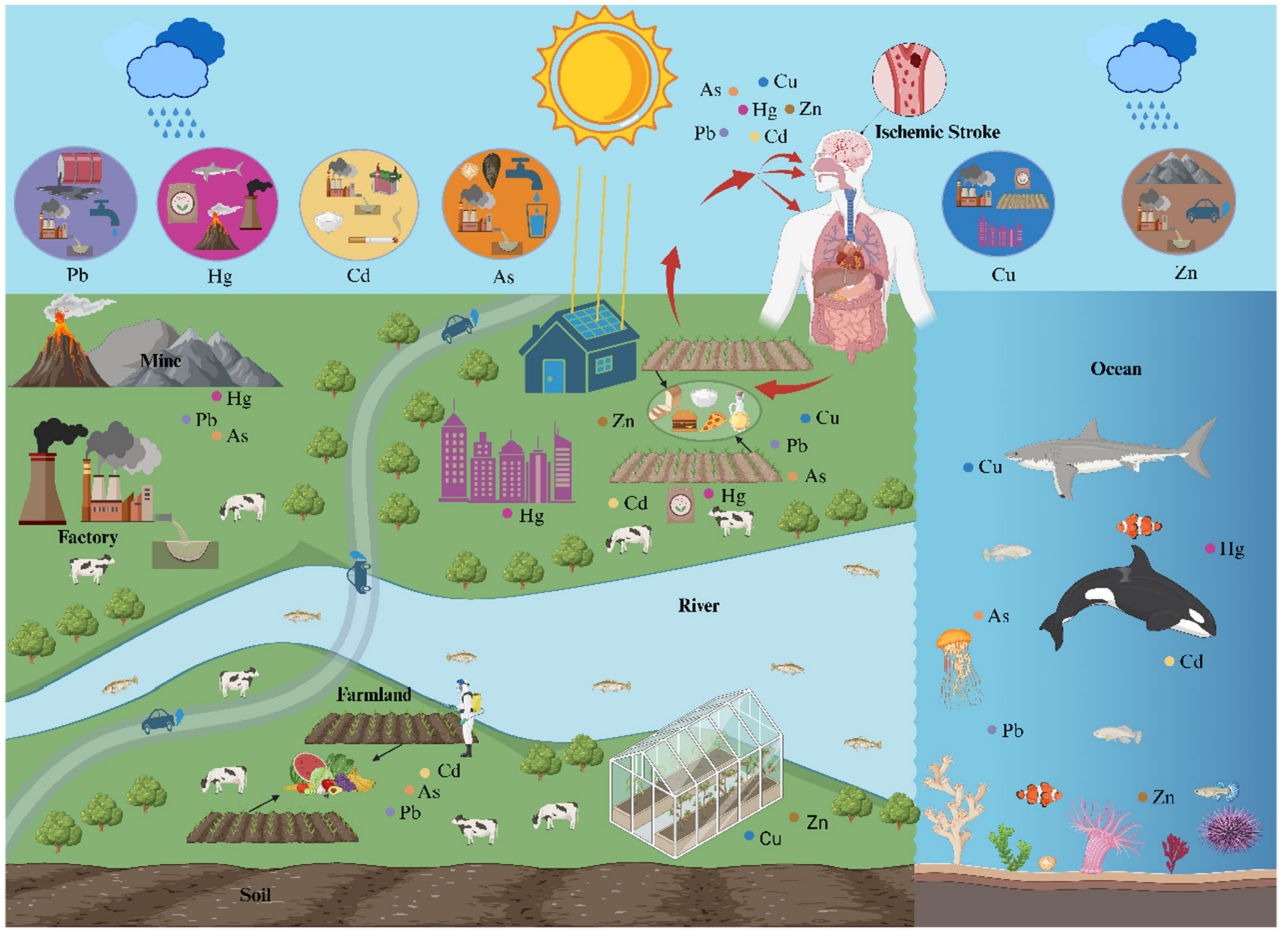
The main sources, contamination pathways, and food chain exposure routes of heavy metals. The diagram depicts the distribution of lead (Pb), mercury (Hg), cadmium (Cd), arsenic (As), copper (Cu), and zinc (Zn) in the nature (rivers, oceans, air, and soil) and the exposure source (agricultural and industrial products). When exposure to heavy metals, they enter the body through inhalation, ingestion and dermal contact, eventually lead to ischemic stroke.

Heavy metals, due to their persistence and bioaccumulation, pose significant risks to health and the environment through various pathways, including natural, industrial, agricultural, and domestic sources, and via food chain exposure (Figure 1). In recent years, numerous studies have confirmed an association between heavy metal exposure and ischemic stroke, with significant variations in the safe exposure thresholds and toxicity limits of different heavy metals in biological samples (see Table 1 for specific reference values).

**Table 1:** Safe and toxic levels in blood and urine of metals.

Metal	Blood		Urine	
	Normal	Toxic	Normal	Toxic
Pb	<5 μg/dL (Ad) <3.5 μg/dL (Ch)	≥10 μg/dL (Ad) ≥5 μg/dL(Ch)	Nm	≥30 μg/gCr
Cd	<0.5 μg/L (Ns) <1 μg/L (S)	≥5 μg/L	<0.5 μg/gCr	≥2 μg/gCr
As	<10 μg/L	Nm	<10 μg/L	Tot-As ≥ 50 μg/L (Ac) Tot-As ≥ 35 μg/gCr (Chr) Inorg-As ≥ 10 μg/L
Hg	Tot-Hg < 1 μg/L MeHg<1.2 μg/L	Tot-Hg ≥ 5 μg/L MeHg ≥ 5.8 μg/L	Inorg-Hg < 3 μg/gCr	Inorg-Hg ≥ 20 μg/gCr
Cu	70–140 μg/dL	≥160 μg/dL	<30 μg/24 h	≥60 μg/24 h
Zn	50–120 μg/dL	≥200 μg/dL	300–600 μg/24 h	>1,000 μg/24 h

The chart summarizes the normal reference ranges and toxicity thresholds for lead, cadmium, arsenic, mercury, copper, and zinc in blood and urine, as provided by the centers for disease control and prevention (CDC), https://wwwn.cdc.gov/TSP/index.aspx. Ac, acute; Ad, adults; As, arsenic; Cd, cadmium; Ch, children; Chr, chronic; Cr, creatinine; Cu, copper; Hg, mercury; Inorg-As, inorganic arsenic; Inorg-Hg, inorganic mercury; MeHg, methyl mercury; Nm, not mention; Ns, Nonsmoker; Pb, lead; S, smoker; Tot-As, total arsenic; Tot-Hg, total mercury; Zn, zinc.

### 2.1 Lead (Pb)

Pb contamination stems from multifaceted anthropogenic activities, encompassing industrial processes (smelting, Pb-acid battery production) (16), mining operations (lead-zinc ore extraction with wastewater effluents) (17), and agricultural utilization of Pb-contaminated pesticides (18). Emerging environmental vectors, such as residues from perovskite solar cell degradation (19), further intensify exposure risks. While inorganic Pb persists predominantly in soil and dust matrices, organic Pb compounds demonstrate heightened bioaccumulation potential due to lipid solubility, though epidemiological investigations primarily focus on inorganic forms given their ubiquity in environmental reservoirs. Population-level studies identify critical exposure routes: inhalation of industrial airborne particulates, ingestion of Pb-contaminated preserved foods (notably canned goods with compromised packaging), and dermal contact with legacy Pb-based paints (20, 21). Recent research indicates that smokers or alcohol consumers have notably higher serum Pb levels than non-smokers and non-drinkers, with this disparity being particularly pronounced in patients with acute ischemic stroke (AIS). This may be attributed to smoking and drinking, which can either directly introduce Pb into the body or affect its metabolism and excretion (22). Moreover, a significant association exists between long-term Pb exposure and an increased risk of stroke (23), as evidenced by a study in the glass production-polluted area of southeastern Sweden: the average urinary Pb concentration (U–Pb) in stroke cases was 0.65 μg/g creatinine, which was significantly higher than that in the control group (0.45 μg/g creatinine) (*p* < 0.01) (7).

### 2.2 Mercury (Hg)

Hg contamination arises from dual pathways encompassing natural geochemical processes and anthropogenic interventions. Natural emissions (≈5,207 mg/yr) originate principally from volcanic degassing, lithospheric weathering cycles, and marine biogenic volatilization (24). At the same time, human-made emissions (2,320 mg/yr) mostly come from three main areas: burning fossil fuels for energy, smelting non-ferrous metals and extracting gold by hand, and using chemicals in agriculture to make Hg-containing fertilizers (25). This human-driven Hg flux has precipitated a 30-fold acceleration in global depositional rates compared to pre-industrial baselines (26). Elements of Hg and inorganic species (like HgCl₂ and HgO) can persist in the environment for a long time. Methylmercury (MeHg), on the other hand, is the main organic form and has amazing biomagnification and neurotoxic properties. This organic speciation predominates in aquatic food chains, constituting the primary exposure vector for human populations (27). A meta-analysis found that Hg exposure is associated with an increase in all-cause mortality and CVD mortality, although the relationship with stroke is not significant (28). Another study, however, did not observe a significant association between serum Hg concentrations and stroke risk but observed a trend of decreasing stroke incidence with increasing Hg levels in women (29).

### 2.3 Cadmium (Cd)

Cd has multiple sources, which include both natural weathering and human activities. Among these, industrial emissions, such as those from mining, metal smelting, and battery production, are significant contributors to Cd pollution (30, 31). Agricultural activities, including the application of phosphate fertilizers, wastewater irrigation, and the use of sewage sludge, also represent major anthropogenic sources of contamination (32). Plasma concentrations of Cd, Pb, and chromium are significantly higher in smokers compared to non-smokers (33). There are different kinds of Cd in the environment, but the soluble Cd ion (Cd^2+^), is the one that gets the most attention because it is so toxic and easy for living things to absorb. Higher levels of Cd in the blood have been linked to a higher risk of an AIS in patients in southern Taiwan, and higher levels of Cd in the urine are also linked to a higher risk of stroke (34). There is a significant dose-dependent positive correlation between blood Cd concentration and the risk of ischemic stroke: among 2,664 American adults, the incidence risk in the population with blood Cd ≥ 0.56 μg/L was 2.67 times that in the group with the lowest concentration (<0.22 μg/L) (odds ratio, OR = 2.67, 95% confidence interval, CI: 1.10–6.49) (35). The association between long-term Cd exposure and stroke risk is significant (relative risk 1.30) (23), especially in southern China, where dietary Cd exposure has a substantial impact on the burden of stroke (36). Another study showed that the blood Cd concentration in patients with AIS was 1.27 ± 0.42 μg/L, significantly higher than that in the control group (0.44 ± 0.16 μg/L, *p* < 0.001). Moreover, the molar ratios of Cd/Zn and Cd/Pb were abnormally elevated, suggesting that Cd imbalance may be involved in the pathogenesis of AIS (37).

### 2.4 Arsenic (As)

The natural sources of As primarily include rock weathering, volcanic activity, and the release of naturally occurring As from groundwater (37). Among anthropogenic sources, the extraction and smelting of As-containing minerals, coal combustion, as well as the use of As-based pesticides and fertilizers in agriculture, wastewater irrigation, and irrigation with As-rich groundwater are the major contributors to pollution (38–40). The global population exposed to hazardous levels of As in groundwater is estimated to be between 94 million and 220 million (41). Both inorganic and organic forms of As exist in the environment, with the inorganic form being more toxic to human health. Inorganic As predominantly occurs as arsenite (iAsIII) and arsenate (iAsV) in food, drinking water, and industrial effluents (42, 43). Natural sources of arsenite include: As-containing rock weathering releasing into groundwater, as well as volcanic activity and soil microbial transformation. Organic As, like arsenobetaine in seafood, is not usually thought to be harmful, but inorganic As may increase the risk of having a stroke. These inorganic As species and their metabolites may contribute to cardiovascular damage. Studies suggest that As can increase the risk of stroke by promoting atherosclerosis, elevating blood pressure, and triggering inflammatory responses (22). A study in Sweden’s “Glass Kingdom” region showed that the average blood As concentration in stroke cases was 2.5 μg/L, significantly higher than 1.9 μg/L in the control group (*p* < 0.01), suggesting that long-term As exposure may increase the risk of stroke (7). Epidemiological studies have further confirmed the association between high As exposure and ischemic stroke: in a study of 1,277 case–control pairs in Shenzhen, China, the risk of ischemic stroke in the highest quartile of plasma As (>2.40 μg/L) was significantly higher than that in the lowest quartile (<0.66 μg/L), with an adjusted OR of 1.88 (95% CI: 1.25–2.81), showing a non-linear dose–response relationship (44); a cohort study of 61,074 adults in Bangladesh showed that the risk of death from ischemic stroke in those with drinking water As ≥50 μg/L was 35% higher than in those with <10 μg/L (hazard ratio, HR = 1.35), and the risk was as high as 72% in women (HR = 1.72) (45). Approximately 100 million people worldwide are threatened by As contamination in drinking water, and Bangladesh has become a worst-hit area due to the largest As poisoning incident in history (46).

### 2.5 Copper (Cu)

The primary sources of Cu pollution include industrial activities such as Cu mining, smelting, and electroplating (47–49); agricultural practices like the use of Cu-based pesticides and fertilizers, as well as wastewater irrigation (50, 51), and urban life and waste disposal (52). The environment contains Cu in various chemical forms, including ionic and complexed states. Its bioavailability is affected by both its chemical form and environmental factors, such as pH and the amount of organic matter present. As an essential trace element, Cu plays a crucial role in several physiological functions; however, excessive intake can pose health risks. Meta-analyses and multiple population studies confirm a significant association between blood Cu concentrations and ischemic stroke risk. A meta-analysis showed that individuals with blood Cu > 117.0 μg/dL had a 72% higher risk than those with <91.2 μg/dL (hazard ratio = 1.72, 95% CI: 1.12–2.65), with a 23% increased risk per 20 μg/dL elevation (OR = 1.23, 95% CI: 1.14–1.33), indicating a clear dose–response relationship (53). A National Health and Nutrition Examination Survey (NHANES) (2011–2016) study of 5,151 adults further validated this: serum Cu > 19.8 μmol/L was linked to a 2.36-fold higher risk vs. <16.4 μmol/L (OR = 2.36, 95% CI: 1.01–5.52), with a 44% higher risk per standard deviation increase (OR = 1.44, 95% CI: 1.11–1.86), showing a linear positive correlation (54). Additionally, elevated Cu levels in acute ischemic stroke patients on admission correlate with poor prognosis (55), and a Chinese community study found a near-linear positive correlation between baseline plasma Cu and first ischemic stroke risk, supporting serum Cu as a potential risk factor (56). However, some studies suggest that a moderate increase in dietary Cu intake may help reduce the risk of stroke (57).

### 2.6 Zinc (Zn)

Zn pollution primarily originates from industrial emissions (such as mining, smelting, and electroplating) (58, 59), agricultural inputs (such as the use of Zn-containing fertilizers and pesticides) (60, 61), and urban runoff (such as the release of Zn oxide particles from tire wear) (62). Free Zn ions are present in smaller amounts in the body, primarily within the nervous system. Most of the Zn in the body is bound to proteins. Zn plays a critical role in cerebral ischemia. Studies have shown that during brain ischemia, excessive Zn^2+^ ion release and accumulation may Pb to neuronal damage and cell death, thereby increasing the risk of stroke (63). Maintaining Zn homeostasis is therefore crucial for brain function—Zn deficiency may impair endothelial function, indirectly increasing stroke risk, while Zn excess can damage neurons and exacerbate difficulties in post-stroke recovery (64). A meta-analysis revealed that serum/plasma Zn concentrations in patients with ischemic stroke (65.39–113.2 μg/dL) were significantly higher than those in healthy controls (SMD = 0.61, *p* = 0.036), suggesting that elevated Zn levels may be associated with an increased risk of stroke (65). However, the US REGARDS study showed a contrasting trend: as serum Zn concentrations increased from the lowest quartile (≤104.86 μg/dL) to the highest quartile (≥140.39 μg/dL), there was a significant negative correlation with the incidence of ischemic stroke (HR = 0.78, 95% CI: 0.61–0.98, trend test *p* = 0.03), and this association was stronger in women (HR = 0.58, *p* < 0.01) (66).

## 3 Mechanistic insights into the impact of heavy metals on ischemic stroke

### 3.1 Heavy metals and oxidative stress in ischemic stroke

**Figure 2:**
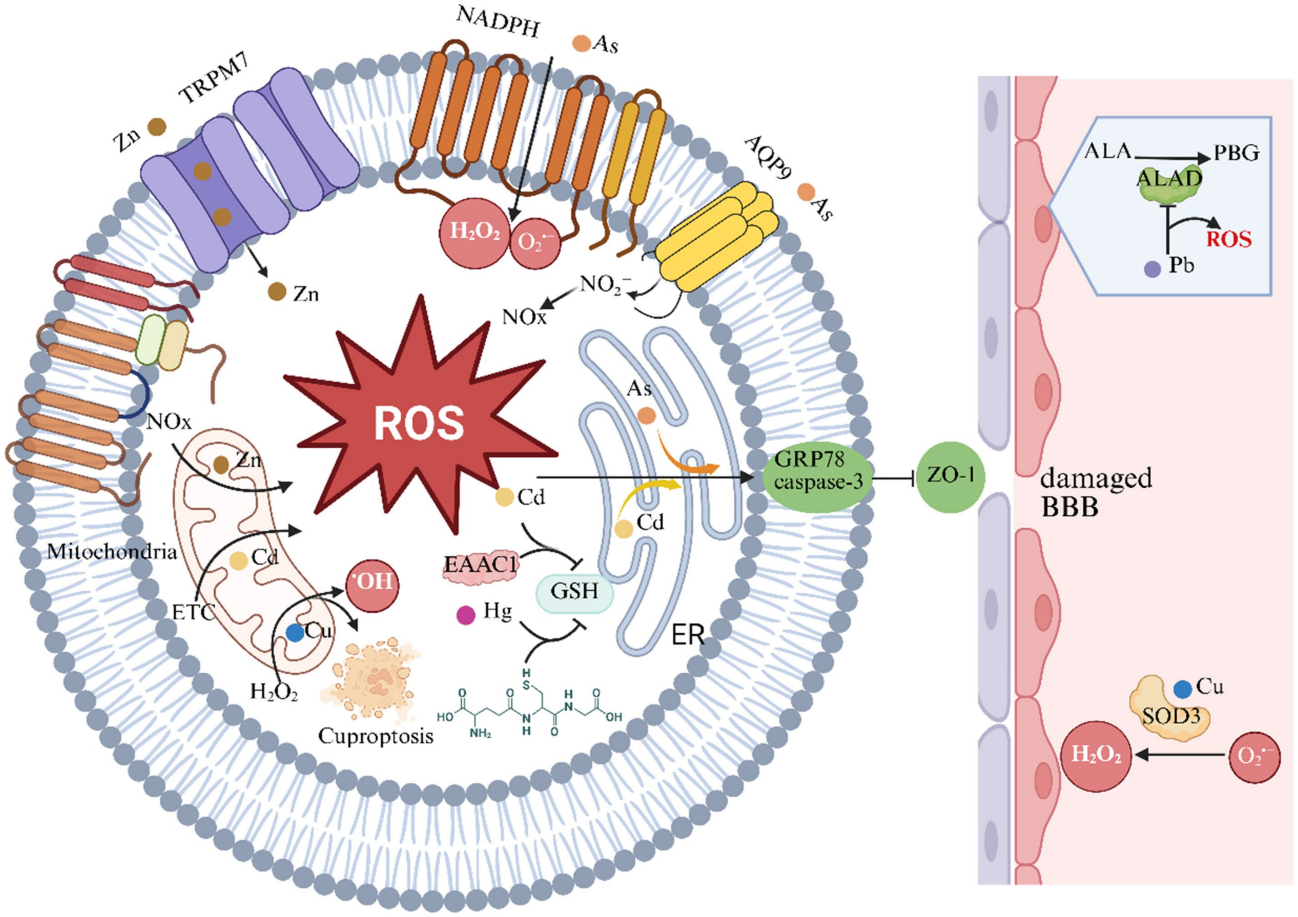
Heavy metals and oxidative stress in ischemic stroke. This chart illustrates the role of heavy metals in the oxidative stress and reactive oxygen species (ROS) generation mechanisms of ischemic stroke. ALAD indicates aminolevulinic acid dehydratase; AQP-9, aquaporin-9; BBB, blood–brain barrier; EAAC1, excitatory amino acid carrier 1; ER, endoplasmic reticulum; ETC, electron transport chain; GSH, glutathione; GRP78, Glucose-Regulated Protein 78; H_2_O_2_, hydrogen peroxide; NADPH, nicotinamide adenine dinucleotide phosphate; NOX, NADPH oxidase; PBG, porphobilinogen; ROS, reactive oxygen species; O_2_^•−^, superoxide anion; SOD, superoxide dismutase; TRPM7, transient receptor potential melastatin 7; ZO-1, zonula occludens-1.

Heavy metals disrupt cellular redox homeostasis through multiple interconnected molecular mechanisms, synergistically inducing oxidative stress and neurotoxicity (Figure 2).

#### 3.1.1 ROS generation and antioxidant defense disruption

Multiple heavy metals converge on redox imbalance through distinct yet interconnected molecular pathways. As (III) (67, 68), zinc ion (Zn^2+^) (69, 70), and Cu (71, 72) exhibit dual roles in both ROS generation and antioxidant system impairment. As triggers superoxide anion (O_2_^•–^) and hydrogen peroxide (H2O2) overproduction via NADPH oxidase (NOX) activation (68), while simultaneously disrupting with antioxidant function by inhibiting the activities of superoxide dismutase (SOD), glutathione peroxidase (GPx), and catalase activities (73). Similarly, Zn^2+^ overload during cerebral ischemia amplifies NOX-derived ROS through mitochondrial α-ketoglutarate dehydrogenase inhibition (70) and glutathione (GSH) reductase suppression (74), creating a self-perpetuating oxidative cascade. Cu’s redox cycling via Fenton reactions generates hydroxyl radicals (71), compounded by its ability to displace iron from cytochrome c oxidase, disrupting mitochondrial redox homeostasis (75). This multi-metal assault on antioxidant defenses creates a “perfect storm” for ED and neuronal apoptosis (76, 77).

#### 3.1.2 Mitochondrial dysfunction and organelle-specific oxidative cascades

Heavy metals target subcellular compartments with striking specificity. Cd^2+^ induces mitochondrial permeability transition pore opening, depleting ATP and amplifying ROS through electron transport chain uncoupling (78). Zn exhibits biphasic mitochondrial interactions—initial protective sequestration followed by pathological accumulation inhibiting complex III and promoting H_2_O_2_ leakage (14). Cu exerts unique proteotoxic stress by displacing iron from mitochondrial Fe-S clusters, triggering Cu-specific cell death (cuproptosis) through lipoylated protein aggregation (72). These organelle-specific mechanisms converge on endoplasmic reticulum (ER) stress, as demonstrated by As- and Cd-induced GRP78 upregulation and caspase-3 activation, ultimately disrupting blood–brain barrier (BBB) integrity through ZO-1 degradation (77, 79).

#### 3.1.3 Thiol reactivity and glutathione system hijacking

Mercaptophilic metals (Pb, Hg, Cd) exploit cellular thiol metabolism for oxidative sabotage. Pb inactivates *δ*-aminolevulinic acid dehydratase (ALAD), causing δ-ALA accumulation and spontaneous ROS generation (80), while MeHg depletes GSH through direct -SH group binding (81). Cd’s inhibition of EAAC1 glutamate transporters reduces cysteine availability for GSH synthesis (79), creating a tripartite attack on the brain’s primary antioxidant system. This thiol-targeted strategy not only increases lipid peroxidation (evidenced by elevated MDA) (82, 83) but also potentiates amyloidogenic processing through NO-mediated vascular dysfunction (80, 84).

#### 3.1.4 Metal transporter dysregulation and redox signaling crosstalk

Emerging evidence reveals metal-specific transport mechanisms modulating oxidative outcomes. Cu’s vascular protection via SOD3 requires Cav-1-mediated stabilization of ATP7A transporters (76), whereas Zn’s neurotoxicity involves TRPM7-mediated neuronal uptake during ischemia (74). As upregulates aquaporin-9 (AQP-9) in astrocytes, facilitating arsenite import and subsequent NOX activation (68). These transport systems create spatial regulation of metal-induced oxidative damage—Cu’s extracellular antioxidant role via SOD3 contrasts with its intracellular mitochondrial toxicity (75, 76), while Zn’s synaptic release versus cytoplasmic accumulation dictates its dual neuroprotective/pro-oxidant effects (69, 74).

### 3.2 Heavy metals and inflammation in ischemic stroke

**Figure 3:**
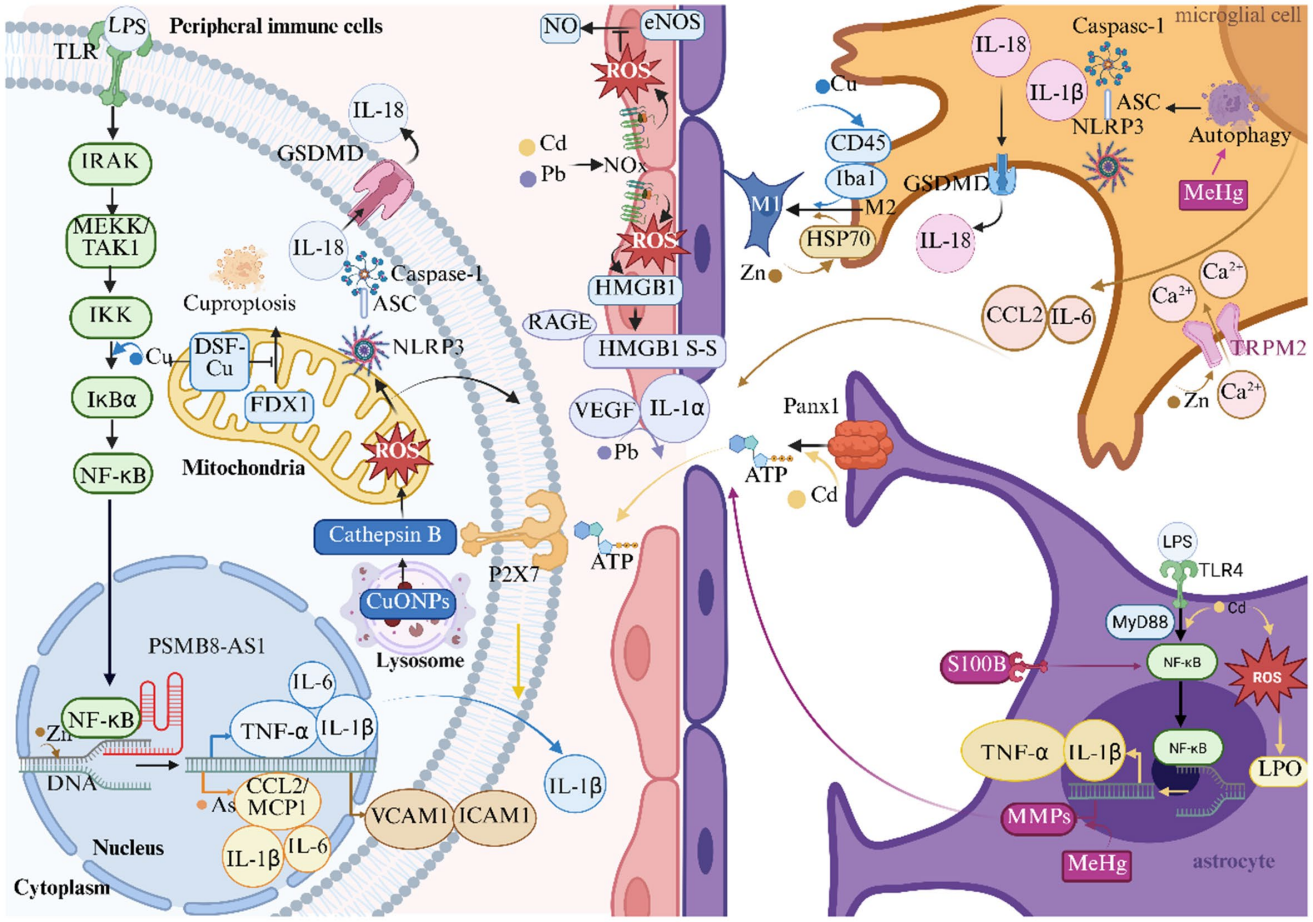
Heavy metals and inflammation in ischemic stroke. This figure illustrates the role of heavy metals in the inflammatory response mechanism of ischemic stroke. ASC Indicates apoptosis-associated speck-like protein containing a card; ATP, adenosine triphosphate; CCL2/MCP1, monocyte chemoattractant protein-1; CuONPs, nanoparticulate copper; DNA, deoxyribonucleic acid; DSF, disulfiram; eNOS, endothelial nitric oxide synthase; FDX1, ferredoxin 1; GSDMD, gasdermin D; HMGB1, high mobility group box 1; HSP70, heat shock protein 70; Iba1, ionized calcium-binding adapter molecule 1; ICAM1, intercellular adhesion molecule 1; IKK, IκB kinase; IL-1α, interleukin-1α; IL-1β, interleukin-1β; IL-6, interleukin-6; IL-18, interleukin-18; IRAK, interleukin-1 receptor-associated kinase; LPO, lipid peroxidation; LPS, lipopolysaccharide; MeHg, methylmercury; MEKK, mitogen-activated protein kinase kinase kinase; TAK1, TGF-β-activated kinase 1; MMPs, matrix metalloproteinases; MyD88, myeloid differentiation primary response 88; NLRP3, NOD-like receptor thermal protein domain associated protein 3; NF-κB, nuclear factor κ-light-chain-enhancer of activated B cells; NO, nitric oxide; PSMB8, proteasome subunit β8; AS1, antisense long non-coding RNA 1; RAGE, receptor for advanced glycation end-products; S-S, disulfide bonds; TLR, toll-like receptor; TLR4, toll-like receptor 4; TNF-α, tumor necrosis factor-α; TRPM2, transient receptor potential melastatin 2; VCAM1, vascular cell adhesion molecule-1; VEGF, vascular endothelial growth factor.

Heavy metal ions exacerbate cerebral ischemic injury by mediating neuroinflammatory responses through multiple mechanisms (Figure 3).

#### 3.2.1 NF-κB-mediated inflammatory cascades

Cu and Zn exhibit dual regulatory roles through NF-κB pathway activation. Cu^2+^ overload in macrophages stimulates IKK-mediated phosphorylation of IκBα, triggering NF-κB nuclear translocation and subsequent upregulation of IL-1β, TNF-α, and IL-6 expression (85). This pro-inflammatory environment makes it easier for leukocytes to enter the ischemic penumbra by increasing the ability of macrophages to phagocytose and the permeability of blood vessels. Zn^2+^ exerts similar effects via PSMB8-AS1-mediated transcriptional regulation, amplifying VCAM1/ICAM1 expression and endothelial adhesion molecule presentation (86). Paradoxically, Cu^2+^ chelation using disulfiram (DSF) suppresses FDX1-mediated cuproptosis pathways, attenuating NF-κB activation and preserving BBB integrity during cerebral ischemia (87). Cd synergistically amplifies this pathway through TLR4/MyD88 signaling, inducing astrocytic NF-κB phosphorylation and subsequent TNF-α/IL-1β release, thereby establishing sustained neuroinflammation (88).

#### 3.2.2 NLRP3 inflammasome activation

Nanoparticulate Cu (copper oxide nanoparticles, CuONPs) initiates biphasic inflammasome activation through lysosomal destabilization and cathepsin B release, generating mitochondrial ROS that prime NLRP3 assembly (89). This mechanism converges with MeHg-induced autophagic stress in microglia, where impaired mitophagy triggers ASC speck formation and caspase-1-dependent IL-1β maturation (90). By creating gasdermin D pores, both metals increase the body’s production of IL-18. This process sets off feedforward loops that keep inflammation going after an ischemic event.

#### 3.2.3 Cytokine/chemokine network dysregulation

As establishes chronic low-grade inflammation via epigenetic reprogramming of circulating lymphocytes, elevating IL-1β, IL-6, and CCL2/MCP1 levels that facilitate monocyte-endothelial interactions (91). Pb exposure in occupational cohorts demonstrates analogous effects through Vascular Endothelial Growth Factor (VEGF)-mediated endothelial activation and IL-1α-driven vascular remodeling (7). Zn^2+^ potentiates this cascade through TRPM2-mediated Ca^2+^ influx in microglia, enhancing IL-6/CCL2 paracrine signaling that disrupts neurovascular units (92).

#### 3.2.4 Glial-immune crosstalk

Cu (II) orchestrates dynamic microglial polarization through CD45/Iba1 modulation, shifting M2 reparative phenotypes toward pro-inflammatory M1 states during ischemia (93). Zn synergistically enhances this transition via HSP70-mediated stress signaling, while MeHg induces S100B-overexpressing reactive astrocytes that secrete matrix metalloproteinases (MMPs) to degrade BBB components (94, 95). Cd^2+^ further compromises neurovascular integrity through PANX1-mediated ATP release, activating P2X7 receptors on perivascular macrophages to sustain IL-1β-dominated inflammation (96, 97).

#### 3.2.5 Oxidative-inflammatory nexus

Pb and Cd establish redox-inflammatory coupling through NOX-derived superoxide generation. This not only inactivates endothelial nitric oxide synthase (eNOS) but also oxidizes HMGB1 to its disulfide form, enhancing RAGE receptor activation on cerebral endothelium (79, 83). Cd^2+^ induces oxidative stress and inflammatory responses, activating the generation of ROS and LPO, promoting the activation of glial cells and neuronal apoptosis, ultimately leading to neural damage (98).

### 3.3 Heavy metals and ED in ischemic stroke

**Figure 4:**
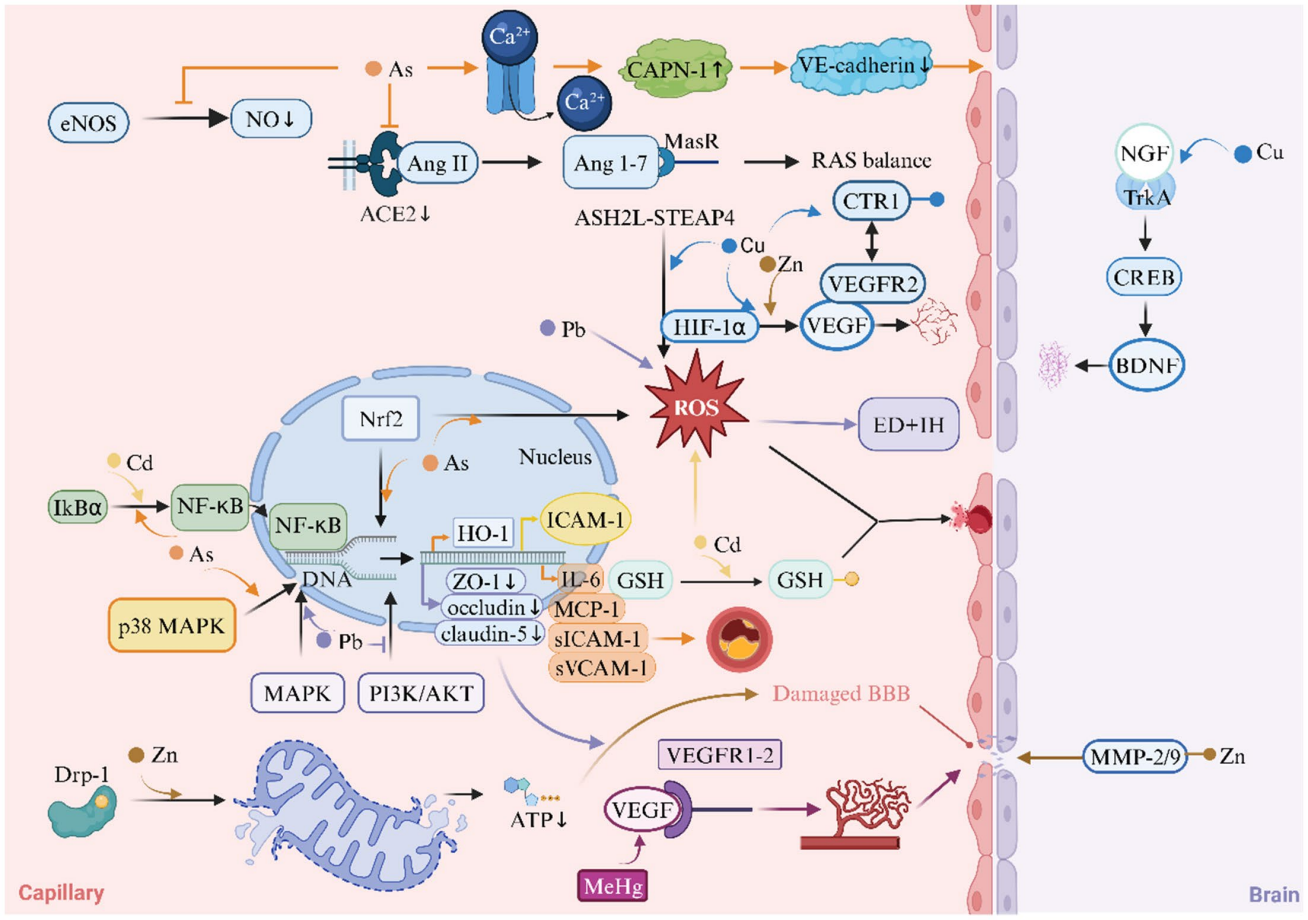
Heavy metals and Endothelial Dysfunction (ED) in ischemic stroke. This diagram illustrates the role of heavy metals in the mechanism of endothelial dysfunction of ischemic stroke. ACE2, angiotensin-converting enzyme 2; AKT, protein kinase B; ASH2L, absen small or homeotic-like 2; BBB, blood–brain barrier; BDNF, brain-derived neurotrophic factor; CAPN-1, calpain-1; CREB, cAMP response element-binding Protein; CTR1, copper transporter 1; Drp1, dynamin-related protein 1; eNOS, endothelial nitric oxide synthase; HIF-1α, hypoxia-inducible factor-1α; HO-1, heme oxygenase-1; ICAM1, intercellular adhesion molecule 1; IH, intimal hyperplasia; IL-6, interleukin-6; MAPK, mitogen-activated protein kinase; MasR, mas receptor; MCP-1, monocyte chemoattractant protein-1; MeHg, methylmercury; MMP, matrix metalloproteinase; NF-κB, nuclear factor κ-light-chain-enhancer of activated B cells; NGF, nerve growth factor; NO, nitric oxide; PI3K, phosphoinositide 3-kinase; RAS, renin-angiotensin system; ROS, reactive oxygen species; sICAM-1, soluble intercellular adhesion molecule-1; STEAP4, six-transmembrane epithelial antigen of prostate 4; sVCAM-1, soluble vascular cell adhesion molecule-1; VEGF, vascular endothelial growth factor; VEGFR2, vascular endothelial growth factor receptor 2; ZO-1, zonula occludens-1.

Heavy metals induce endothelial dysfunction and blood-brain barrier disruption through multiple mechanisms, including redox imbalance, mitochondrial dysfunction, and immune-inflammatory activation (Figure 4).

#### 3.3.1 Redox imbalance and mitochondrial dysfunction

Heavy metals orchestrate Endothelial Dysfunction (ED) through redox imbalance and mitochondrial perturbations. Cd induces oxidative stress by depleting GSH reserves and generating ROS, which impair cerebral microvascular endothelial cell (bEnd.3) integrity and exacerbate BBB leakage (99, 100). As (III) similarly elevates ROS levels via Nrf2 pathway activation, paradoxically upregulating heme oxygenase-1 (HO-1) while suppressing eNOS activity, thereby reducing nitric oxide (NO) bioavailability critical for vasodilation (101, 102). Zn buildup in mitochondria makes endothelial cell damage and BBB worse through a Drp1-dependent pathway for mitochondrial fission (103). Cu exhibits dual roles: while excessive Cu uptake in diabetes exacerbates endothelial ROS via the ASH2L-STEAP4 axis (15), controlled Cu delivery enhances VEGF-mediated vascular repair through Cu Transporter 1 (CTR1) -VEGFR2 signaling (104, 105).

#### 3.3.2 Immune activation and adhesion molecule upregulation

Pro-inflammatory signaling constitutes a unifying mechanism across multiple metals. Cd uniquely activates NF-κB via IkBα tyrosine phosphorylation (not degradation), driving ICAM-1 overexpression in bEnd.3 cells and promoting leukocyte-endothelial adhesion (99). As (III) synergistically amplifies inflammation by inducing MCP-1, IL-6, and sICAM-1/sVCAM-1 through p38 MAPK/NF-κB crosstalk, accelerating atherosclerosis and microvascular occlusion (106, 107). Pb increases the production of ROS in endothelial cells and smooth muscle cells, leading to ED and intimal hyperplasia (IH) (108, 109). Notably, Cu (II)'s pro-angiogenic effects via VEGF/BDNF release counterbalance inflammatory damage during stroke recovery (110, 111).

#### 3.3.3 Vascular permeability and barrier dysfunction

BBB breakdown emerges as a critical endpoint across metal toxicities. Pb disrupts the integrity of the BBB by reducing the expression of tight junction proteins (ZO-1, occludin, claudin-5) through the MAPK and PI3K/AKT signaling pathways (112, 113). MeHg induces VEGF/VEGFR1-2 overexpression, causing pathological angiogenesis with leaky vasculature and cerebral edema (114, 115). Cu and Zn promote physiological angiogenesis through CTR1-VEGFR2 interaction and HIF-1α/VEGF activation (104, 116). As (III) exerts dual-barrier disruption through CAPN-1 activation: rapid calcium influx triggers VE-cadherin degradation at adherens junctions, while chronic exposure downregulates ACE2/MasR axis, impairing the protective renin-angiotensin system (117, 118). Zn overload activates matrix metalloproteinases (MMPs) -2/9 via metalloproteinase-Zn interactions, directly digesting basement membrane components and facilitating BBB leakage (119). Cd further destabilizes pericyte-endothelial crosstalk, inducing pericyte contraction and microvascular flow arrest (120).

### 3.4 Heavy metals and neurotransmitter effects in ischemic stroke

**Figure 5:**
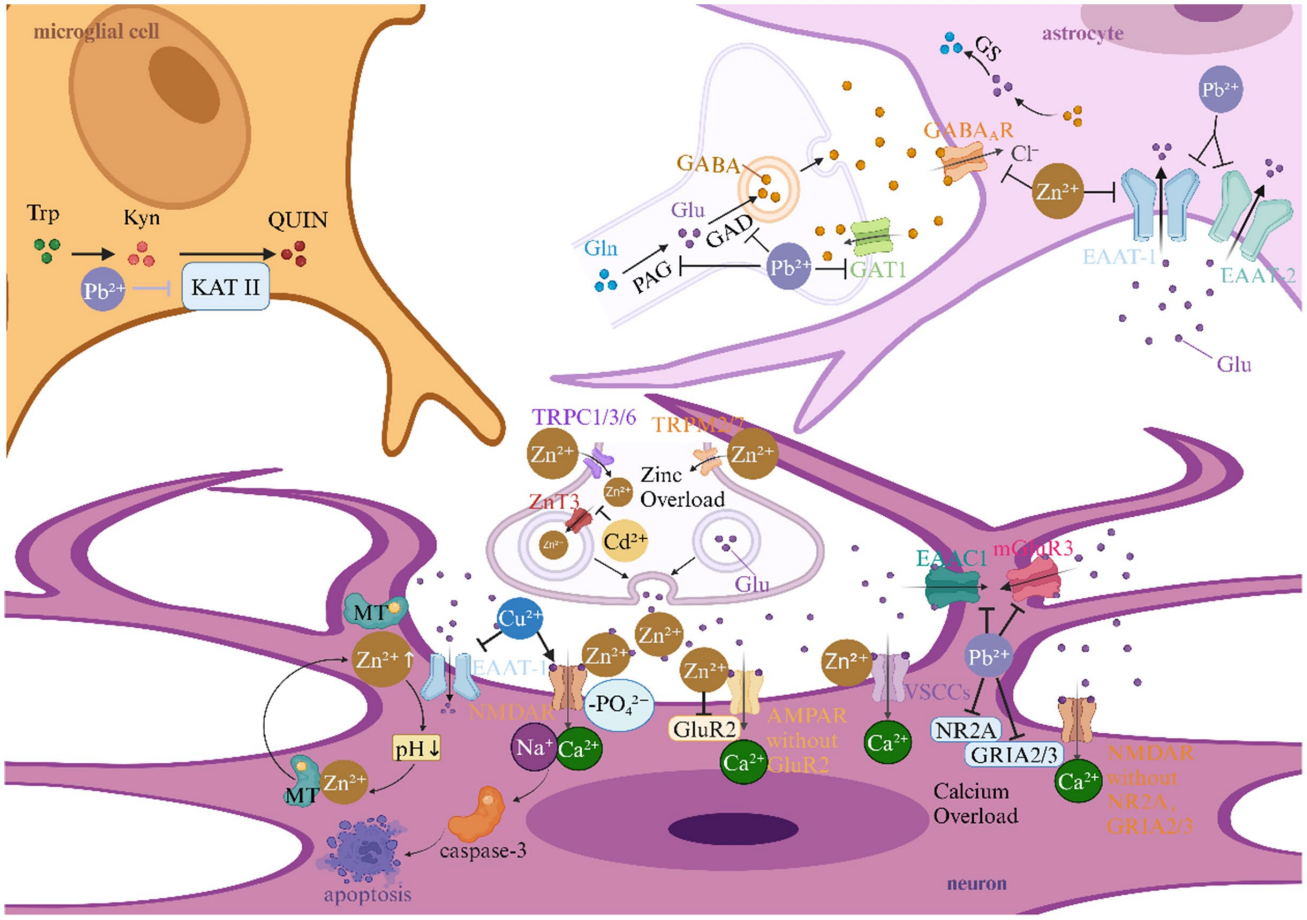
Heavy metals and neurotransmitter effects in ischemic stroke. This picture illustrates the role of heavy metals in the neurotransmitter mechanism of ischemic stroke. AMPAR, α-amino-3-hydroxy-5-methyl-4-isoxazolepropionic acid receptor; EAAC1, excitatory amino acid carrier 1; EAAT, excitatory amino acid transporter; GABA, gamma-aminobutyric acid; GABA_A_R, gamma-aminobutyric acid type A receptor; GAD, glutamic acid decarboxylase; GAT1, gamma-aminobutyric acid transporter 1; Glu, Glutamate; GluR2, α-amino-3-hydroxy-5-methyl-4-isoxazolepropionic acid receptor subunit; GLAST, glutamate aspartate transporter; GLS, glutaminase; GLT-1, glutamate transporter 1; GRIA2/3, α-amino-3-hydroxy-5-methyl-4-isoxazolepropionic acid receptor subunits; GRM3, glutamate receptor 3; GS, Glutamine Synthetase; KAT II, kynurenine aminotransferase II; Kyn, kynurenine; mGluR3, metabotropic glutamate receptor 3; NMDA, N-methyl-D-aspartate; NMDAR, N-methyl-D-aspartate receptor; NR2A, fast synaptic N-methyl-D-aspartate receptor subunit; QUIN, quinolinic acid; Trp, tryptophan; TRP, transient receptor potential; TRPC, transient receptor potential canonical; TRPM, transient receptor potential melastatin; VSCCs, voltage-sensitive calcium channels; ZnT3, zinc transporter 3.

Heavy metals exacerbate excitotoxicity and neuronal injury following ischemia by disrupting synaptic metal ion homeostasis and neurotransmitter systems (Figure 5).

#### 3.4.1 Glutamatergic excitotoxicity amplification via synaptic Zn overload

The interplay between Zn dyshomeostasis and glutamatergic signaling constitutes a pivotal axis in ischemic neuronal injury. During acute ischemia, synaptic vesicles release excessive Zn^2+^ that synergizes with glutamate to activate post-synaptic N-methyl-D-aspartate (NMDA) receptors (NMDARs), Ca^2+^-permeable α-amino-3-hydroxy-5-methyl-4-isoxazolepropionic acid (AMPA) receptors (GluR2-lacking), and voltage-sensitive calcium channels (VSCCs), creating a self-perpetuating cycle of cation influx (121–123). Notably, Zn-induced acidification mobilizes metallothionein-bound Zn^2+^ reservoirs, exponentially increasing intracellular free Zn^2+^ concentrations (124). This Zn-glutamate crosstalk extends to transporter modulation: Zn^2+^ inhibits excitatory amino acid transporter-1 (EAAT-1) and GABA_A receptors, while Pb^2+^ downregulates astrocytic GLT-1 and neuronal EAAC1, collectively impairing glutamate clearance (125–127). Crucially, Cd^2+^ exacerbates this cascade by suppressing ZnT3-mediated Zn sequestration in hippocampal neurons, thereby enhancing vulnerability to excitotoxic insults (128).

#### 3.4.2 NMDA receptor hypersensitization and subunit remodeling

Heavy metals cause NMDA receptors to become overactive in two ways: they directly increase the activity of channels and change the make-up of subunits. Cu^2+^ elevates hippocampal glutamate levels while promoting NMDAR phosphorylation, driving caspase-3-mediated apoptosis (129). Concurrently, Zn^2+^ and Pb^2+^ induce transcriptional shifts favoring calcium-permeable receptor variants: Zn^2+^ downregulates GluR2 expression in AMPA receptors, while Pb^2+^ reduces NR2B-containing NMDARs and modifies GRIA2/3 subunit stoichiometry (130–132). These changes caused by metals on receptors create neurotoxic “hotspots” where normal glutamate signaling gets worse and leads to an unhealthy amount of calcium and Zn.

#### 3.4.3 Astrocyte-neuron metabolic coupling disruption

Metallotoxic interference with glial neurotransmitter recycling emerges as a critical stroke amplifier. Pb^2+^ disrupts the glutamate-glutamine cycle by suppressing glutaminase (GLS) activity and GLAST/GLT-1 expression, while paradoxically enhancing K^+^-stimulated glutamate release—a formula for synaptic glutamate spillover (126, 127). The extracellular buildup of glutamate works with metal-induced Zn release from presynaptic terminals to make a neurotoxic feedback loop that is stronger than the brain’s defenses against neuronal death after an ischemic event.

#### 3.4.4 GABAergic inhibition attenuation and kynurenine pathway activation

Heavy metals strategically disarm endogenous neuroprotective systems by targeting inhibitory neurotransmission. Zn^2+^ directly blocks GABA_A receptor chloride currents, diminishing inhibitory postsynaptic potentials during ischemic depolarization waves (133). Pb^2+^ exerts complementary effects by reducing GABA synthesis (via GAD suppression) and enhancing kynurenine aminotransferase II (KAT II) activity, shifting tryptophan metabolism toward neurotoxic quinolinic acid production (131). This dual assault on GABAergic tone and excitatory/inhibitory balance creates a permissive environment for spreading depolarization and infarct expansion.

#### 3.4.5 Voltage-independent cation channel activation

Emerging evidence implicates transient receptor potential (TRP) channels as convergence points for metal neurotoxicity. Ischemia-induced Zn^2+^ influx occurs not only through classical voltage-gated channels but also via TRPC1/3/6 and TRPM2/7 activation, enabling massive cation entry independent of membrane depolarization (134). This pathway synergizes with Pb^2+^-induced metabotropic glutamate receptor 3 (GRM3) downregulation, effectively removing the “molecular brakes” on post-synaptic excitation (132). The resultant cation overload propagates through neuronal networks via gap junctions, exacerbating peri-infarct depolarizations.

### 3.5 Heavy metals and coagulation mechanisms in ischemic stroke

**Figure 6:**
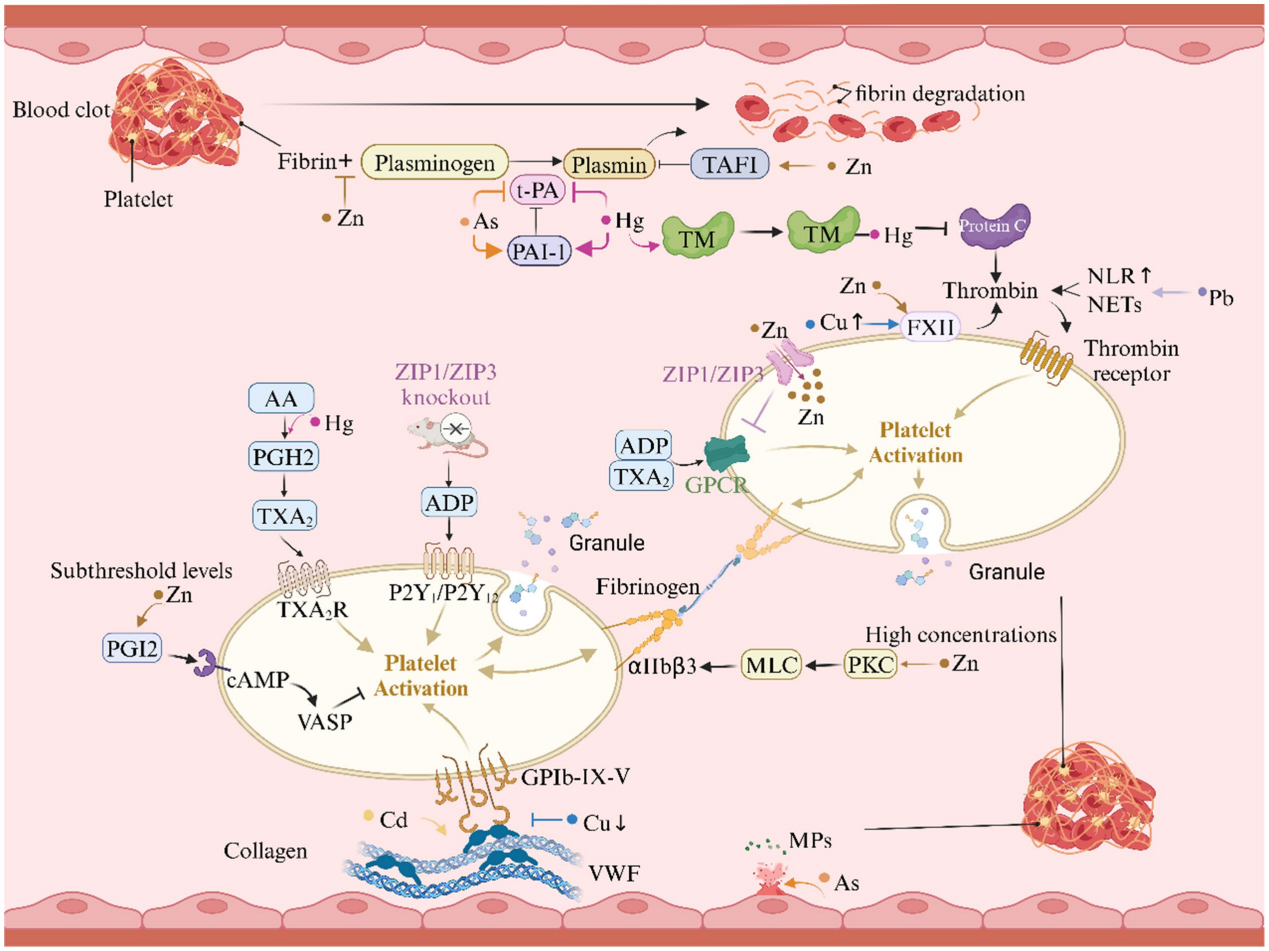
Heavy metals and coagulation mechanisms in ischemic stroke. This graph illustrates the role of heavy metals in the coagulation mechanism of ischemic stroke. AA, arachidonic acid; ADP, adenosine diphosphate; cAMP, cyclic adenosine monophosphate; FXII, factor XII; GPCR, G-protein coupled receptor; GP, glycoprotein; GSH, glutathione; GST, glutathione S-transferase; MLC, myosin light chain; MPs, microparticles; NETs, neutrophil extracellular traps; NLR, neutrophil-to-lymphocyte ratio; PAI-1, plasminogen activator inhibitor-1; PGH₂, prostaglandin H₂; PGI₂, prostacyclin I₂; PKC, protein kinase C; PS, protein S; TAFI, thrombin-activated fibrinolysis inhibitor; TM, thrombomodulin; t-PA, tissue-type plasminogen activator; TXA₂, thromboxane A₂; VASP, vasodilator-stimulated phosphoprotein; vWF, von Willebrand factor; ZIP, zinc and iron-regulated transmembrane proteins.

Heavy metal interfere with platelet function, coagulation, and the fibrinolytic system through various mechanisms, thereby affecting thrombus formation (Figure 6).

#### 3.5.1 Regulation of platelet activation

Hg^2+^ stops platelets from quiescently forming by blocking Na^+^-K^+^-ATPase activity through GSH/GST-mediated enzyme binding. The effect changes the sodium gradient needed for platelets to rest and encourages them to become active. Furthermore, Hg^2+^ enhancing ADP-induced platelet aggregation by activating the TXA₂/PGH₂ pathway (135–137). Zn^2+^ exhibits a concentration-dependent biphasic effect. At high concentrations, Zn^2+^ triggers full activation of αIIbβ3 integrins and promotes platelet aggregation via the protein kinase C (PKC)/myosin light chain (MLC) phosphorylation pathway, whereas at low concentrations, Zn^2+^ inhibits platelet activation and thrombus formation by enhancing prostacyclin I₂ (PGI₂) signaling through the cyclic adenosine monophosphate (cAMP)/vasodilator-stimulated phosphoprotein (VASP) pathway (138–140). Additionally, ZIP1/ZIP3 transporters play a critical role in maintaining Zn^2+^ homeostasis in platelets, and their deficiency leads to hyperactive G protein-coupled receptor (GPCR) signaling, accelerating thrombus formation (141).

#### 3.5.2 Interference with the coagulation cascade

Cd, for instance, promotes excessive expression of von Willebrand factor (vWF) in endothelial cells, thereby enhancing platelet adhesion under shear stress (142). Cu imbalance presents a paradox in thrombus formation: Cu deficiency reduces the binding ability of vWF to platelets, while excess Cu alters platelet surface charge and activates factor XII (FXII), thereby accelerating coagulation (143–145). Pb^2+^ elevate the neutrophil-to-lymphocyte ratio (NLR), promoting the formation of neutrophil extracellular traps (NETs) that enhance thrombin generation, further exacerbating thrombus formation (146, 147).

#### 3.5.3 Fibrinolysis inhibition and microparticle exposure

As ions inhibit thrombus dissolution by downregulating tissue-type plasminogen activator (t-PA) expression and upregulating plasminogen activator inhibitor-1 (PAI-1) expression, thereby generating antiprotease thrombi (148). Zn ions, by competitively inhibiting plasminogen binding to histidine-rich domains, enhance the activity of thrombin-activated fibrinolysis inhibitor (TAFI), thereby suppressing fibrinolysis and promoting thrombus formation (149, 150). Hg, by inhibiting the thrombomodulin (TM)/protein C and t-PA/PAI systems, reduces anticoagulant activity and suppresses fibrinolysis, resulting in a procoagulant state (151). Moreover, As₂O₃ induces exposure of phosphatidylserine (PS) on endothelial cells and generates microparticles (MPs) carrying PS, thereby enhancing coagulation activity and further aggravating coagulation abnormalities (138, 152).

### 3.6 Synergistic, antagonistic, or additive effects of combined heavy metal exposure

#### 3.6.1 Synergistic effects of combined heavy metal exposure

Cu overload can stimulate IKK-mediated IκBα phosphorylation in macrophages, promoting NF-κB nuclear translocation and upregulating the expression of inflammatory factors such as IL-1β, TNF-α, and IL-6 (85); Cd synergistically amplifies this pathway through the TLR4/MyD88 signaling pathway, inducing NF-κB phosphorylation in astrocytes and releasing TNF-α/IL-1β, thereby triggering persistent neuroinflammation (88). The two synergistically activate the NF-κB pathway to enhance neuroinflammation, creating a strong pro-inflammatory environment. They facilitate the entry of leukocytes into the ischemic penumbra by improving macrophage phagocytosis and increasing vascular permeability, significantly elevating the risk of stroke. In addition, As and Hg jointly inhibit the glutathione system to exacerbate oxidative stress, increasing reactive oxygen species production by 30–50% compared with individual exposures, collectively reflecting the synergistic effect of combined heavy metal exposure (153).

#### 3.6.2 Antagonistic effects of combined heavy metal exposure

Zn can antagonize Cu overload-induced neurodamage by maintaining metal ion homeostasis in the body: on one hand, it reduces Cu entry into neurons by competing for the transporter CTR1 and regulates cuproptosis (154, 155); on the other hand, as a cofactor of SOD, it enhances its activity and reduces Cu-mediated ROS production (156). The two exhibit an antagonistic effect through transport competition, regulation of cell death, and balance of the antioxidant system.

#### 3.6.3 Additive effects of combined heavy metal exposure

Pb reduces the expression of tight junction proteins via the MAPK and PI3K/AKT pathways (113); Cd damages endothelial cells through oxidative stress, disrupts pericyte-endothelial interactions, and activates the NF-κB pathway (79, 99, 120); Zn overload impairs the BBB via Drp1-dependent mitochondrial fission and activation of MMP-2/9 (103, 119); As degrades VE-cadherin and downregulates the ACE2/MasR axis by activating CAPN-1 (117); MeHg induces overexpression of VEGF and its receptors, leading to vascular leakage (114). These heavy metals damage the BBB through mechanisms such as disrupting tight junctions, inducing oxidative stress, activating proteases, and interfering with angiogenesis. When co-exposed, these mechanisms act synergistically, potentially resulting in greater BBB disruption than single exposures, exhibiting an additive effect.

## 4 Targeted therapies for heavy metal-related ischemic stroke

### 4.1 Antioxidant

Natural antioxidants safeguard particular tissues from metal-induced neurotoxicity by removing free radicals, regulating redox levels, or increasing the efficacy of endogenous antioxidant enzymes. Tannic acid (TA) reduces the accumulation of Cd^2+^ in the brain by competitively binding to them. At the same time, it selectively increases the activity of catalase (CAT) and GPx in the hippocampus. This significantly reduces oxidative damage in models of chronic Cd/Pb exposure (12). Dansheninone IIA (TSA) ameliorates Pb-induced cognitive impairment by elevating SOD and GSH levels, decreasing malondialdehyde (MDA) concentration, and synergistically enhancing brain-derived neurotrophic factor (BDNF) expression (157). Natural polyphenolic compounds, including hesperidin (HP) and resveratrol, alleviate Cd-induced oxidative stress and synaptic impairment by reestablishing the GSH/non-protein thiol (NP-SH) balance, preventing lipid peroxidation (LPO) and protein carbonylation (PC), directly neutralizing reactive oxygen species (ROS), and improving cytochrome P450 enzymes to reduce neuronal apoptosis (158, 159). Curcumin augments the activity of SOD and GPx by chelating Cu^2+^ and Zn^2+^. It enhances bioavailability by employing mitochondrial approaches such as triphenylphosphonium (TPP) modification, consequently amplifying its antioxidant and anti-apoptotic properties (160, 161).

N-acetylcysteine (NAC) is a synthetic antioxidant that possesses metal chelation and antioxidant properties. The thiol group can directly chelate chromium, Cd, and cobalt ions, inhibiting their intestinal absorption and subsequent neurotoxicity. In studies of Hg or Pb poisoning, NAC helps protect brain cells by balancing GSH levels and reducing malondialdehyde (MDA) concentrations, which can prevent damage to DNA and cell death. Nanoparticle delivery techniques greatly improve the concentration of NAC in the brain (162–164). In addition, 14.7-aminoquinoline derivatives impede the Fenton reaction by Cu chelation and stimulate the Nrf2 pathway, enhancing the transcription of antioxidant enzymes, showing significant metal antagonistic properties (165, 166).

### 4.2 Metal chelators

Metal chelators bind to heavy metal ions in the body, which assists in decreasing their buildup and neurotoxicity. In the ischemia/reperfusion (I/R) injury model caused by middle cerebral artery occlusion (MCAO), the Zn chelator TPEN markedly reduced post-stroke inflammation and neuronal damage. This was achieved by decreasing the expression of pro-inflammatory cytokines (TNF-α, IL-6), obstructing the activation of the NF-κB pathway, and elevating the levels of the anti-inflammatory cytokine IL-10 (13). Peridin, a natural Zn chelator, on the other hand, fought off Zn-induced neurotoxicity and maintained spatial memory function (167). Calcium disodium EDTA (Ca-EDTA), a traditional chelator, has demonstrated the ability to counteract the inhibitory effects of Zn^2+^ on thrombolytic therapy. *In vitro* experiments revealed that it nearly doubled the efficacy of tissue plasminogen activator (tPA), restoring its activity by 35–50%. In *in vivo* models, it significantly enhanced reperfusion rates (from a baseline of 45 to 75–80%) while diminishing the risk of hemorrhage (168, 169). Notably, nano-delivery systems can further enhance the neuroprotective efficacy of chelators. For instance, polysorbate-80-coated polymeric nanoparticles enable efficient delivery of the hexadentate iron chelator desferrioxamine across the BBB, specifically clearing abnormally accumulated iron and Cu ions in the brain. This process inhibits oxidative stress and cuproptosis, thereby achieving precise protection of neurons (170).

### 4.3 Dietary adjustment

Dietary adjustments hold significant intervention value in heavy metal-related ischemic stroke. Studies indicate that Cu, an essential neuromodulator in cerebral ischemia/reperfusion injury, may exacerbate pathological processes by inducing cuproptosis via oxidative stress when in excess, making the regulation of Cu homeostasis a potential therapeutic target (170). Nutritional interventions act through multiple mechanisms: antioxidant nutrients (e.g., B vitamins, glutathione, Zn) alleviate free radical damage and improve neurological recovery; protein supplementation reverses suppressed protein synthesis in ischemic regions, promoting cognitive recovery. Additionally, stroke patients often have insufficient Zn intake, and Zn supplementation can ameliorate neurological deficits (171). These findings support the positive role of diet in regulating heavy metal toxicity, alleviating oxidative stress, and facilitating stroke rehabilitation.

### 4.4 Other neuroprotective strategies

In addition to antioxidant and chelation therapies, interventions targeting inflammatory pathways and epigenetic regulation have also demonstrated neuroprotective potential. For example, MeHg induces inflammatory responses by activating microglial NLRP3 inflammasomes and autophagosomes. The NLRP3 inhibitor MCC950 can simultaneously mitigate MeHg-induced microglial damage and inflammation triggered by Cu ion-activated TLR4 signaling (14). In an As trioxide (ATO) exposure model, the calpain-1 (CAPN-1) inhibitor ALLM exerts vascular protective effects by improving endothelial cell dysfunction (such as increased vascular permeability and abnormal low-density lipoprotein uptake) through the inhibition of CAPN-1 activity (117).

Disulfiram (DSF) has been shown to protect mitochondrial integrity and improve outcomes in cerebral ischemia in mice by downregulating FDX1 to regulate Cu homeostasis, inhibiting the HSP70/TLR4/NLRP3 inflammatory pathway, and reducing oxidative stress (76). Additionally, RNA interference targeting the epigenetic regulator ASH2L (anti-ASH2L short hairpin RNA (shRNA) adeno-associated virus) reduces the expression of Cu transporters (CTR1/STEAP4), thus decreasing Cu uptake by endothelial cells and reversing Cu-induced oxidative stress, inflammation, and vascular dysfunction (15, 104).

RNA interference (RNAi) shows promise as a neuroprotective strategy for improving ischemic stroke by regulating Cu metabolism. As an essential trace element in cerebral ischemia/reperfusion (I/R) injury, Cu can induce oxidative stress and cuprotosis (a Cu-dependent form of cell death) when in excess. RNAi can target Cu transporters (e.g., ATP7A, ATP7B), Cu chaperones (e.g., COX17, CCS), and Cu-related enzymes (e.g., SOD1) to regulate the intracellular distribution and activity of Cu ions. This regulation alleviates oxidative damage, inflammatory responses, and mitochondrial dysfunction, thereby reducing neuronal death and enhancing the therapeutic efficacy in stroke (170).

It should be noted that most of the targeted therapeutic strategies discussed in this section, including the use of antioxidants, metal chelators, dietary adjustments, and other neuroprotective interventions, currently rely mainly on preclinical evidence from animal models and *in vitro* studies. Direct clinical translational data specific to heavy metal-related ischemic stroke remain relatively limited. This is associated with the complexity of environmental exposure patterns, the difficulty in classifying patient subgroups according to heavy metal exposure status, and the fact that research in this field is still in its early stages. However, mechanistic findings from preclinical studies—such as the neuroprotective effects of TA in alleviating Cd-induced oxidative damage, the ability of Ca-EDTA to enhance thrombolysis by chelating Zn, and the role of RNAi in regulating Cu homeostasis—provide sufficient basis for subsequent clinical exploration. They also lay the groundwork for designing targeted clinical trials to verify the therapeutic potential of these strategies in populations with heavy metal-related cerebrovascular damage.

## 5 Conclusion

This review thoroughly examines the pathogenic role of heavy metals (Pb, Cd, As, Hg, Cu, Zn) in ischemic stroke, emphasizing their complex effects via interconnected molecular processes. Heavy metals specifically induce oxidative stress by activating NOX and impairing mitochondrial function; modulate neuroinflammation through the NF-κB/NLRP3 signaling pathway; disrupt endothelial tight junctions, compromising the integrity of the BBB; interfere with glutamatergic neurotransmission via excitotoxicity mediated by Zn/Pb; and disrupt coagulation processes by excessively activating platelets.

Heavy metal intake is a modifiable risk factor for stroke, according to this innovative study that combines molecular neurology with environmental toxicology. Researchers should devote more time to studying translatable approaches so they can develop non-invasive biomarkers for early stroke risk assessment, target metal transport proteins, and improve our pharmacological understanding of metal transporters. Researchers should also look at community-based initiatives to lower metal exposure in the environment and the spatiotemporal modeling of metal-induced harm. These multi-target treatments consider the neurovascular architecture changes over time in addition to the short-term impacts of metal poisoning.

Heavy metal ingestion is a modifiable risk factor for stroke, as shown by many pieces of evidence presented in this novel review that innovatively combines molecular neurology with environmental toxicology. Strategies oriented toward public health intervention should be the primary goal of future studies. This includes developing non-invasive biomarkers to assess the risk of stroke at an early stage, identifying and pharmacologically modifying metal transport proteins, and expanding our understanding of metal transporters. Research on community-based initiatives that decrease metal exposure in the environment and spatial–temporal modeling of metal-induced harm are also warranted. The results of this study provide important information for the design of long-term public health programs and the mitigation of heavy metal-induced cerebrovascular damage.
